# Association Between Serum Follicle‐Stimulating Hormone Levels and Risk of Diabetes in Middle‐Aged Men: A Long‐Term Population‐Based Longitudinal Study

**DOI:** 10.1002/edm2.70262

**Published:** 2026-07-06

**Authors:** Maryam Mousavi, Faeghe Firouzi, Fahimeh Ramezani Tehrani, Fereidoun Azizi, Samira Behboudi‐Gandevani

**Affiliations:** ^1^ Reproductive Endocrinology Research Center, Research Institute for Endocrine Molecular Biology, Research Institute for Endocrine Sciences Shahid Beheshti University of Medical Sciences Tehran Iran; ^2^ Foundation for Research & Education Excellence Vestavia Hills Al USA; ^3^ Endocrine Research Center, Research Institute for Endocrine Disorders, Research Institute for Endocrine Sciences Shahid Beheshti University of Medical Sciences Tehran Iran; ^4^ Faculty of Nursing and Health Science Nord University Bodø Norway

**Keywords:** follicle‐stimulating hormone, men, Tehran lipid and glucose study, type 2 diabetes mellitus

## Abstract

**Aim:**

This study aimed to investigate the association between serum FSH levels and both the prevalence and incidence of T2DM among middle‐aged and older men.

**Methods:**

This study utilised data from 1097 men aged ≥ 40 years, who participated in the population‐based Tehran Lipid and Glucose Study. Participants were followed over six phases between 2002 and 2021. FSH levels were measured at baseline, and participants were followed for incident T2DM over 3774.78 person‐years. Multivariable logistic and Cox regression models were used to examine associations between FSH levels and prevalent and incident T2DM, adjusting for demographic and metabolic confounders. Subgroup analyses were performed by age group (< 50 vs. ≥ 50 years).

**Results:**

At baseline, the prevalence of T2DM was 18.41%, and no significant association was observed between FSH levels and T2DM prevalence after full adjustment (OR: 1.0; 95% CI: 0.98–1.03). During follow‐up, 302 new T2DM cases were identified. In the fully adjusted Cox model, higher FSH levels were independently associated with a lower risk of developing T2DM (HR: 0.96; 95% CI: 0.93–0.99). The inverse association was especially evident in men aged ≥ 50 years (HR: 0.96; 95% CI: 0.93–0.99), whereas no significant association was found in younger men.

**Conclusion:**

Higher serum FSH levels were independently associated with a reduced risk of incident T2DM in men, particularly among those aged ≥ 50. These findings suggest a potential protective metabolic role of FSH in older men. Further research is warranted to elucidate the non‐reproductive roles of FSH in men.

## Graphical abstract

Higher baseline serum FSH levels were independently associated with a lower risk of incident type 2 diabetes in middle‐aged and older men over long‐term follow‐up. This inverse association was strongest in men aged ≥ 50 years and those with elevated FSH/LH ratios, suggesting a protective endocrine‐metabolic role of FSH.

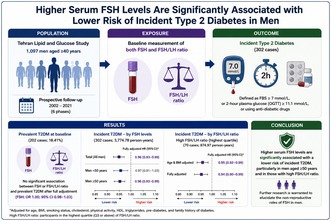

## 1 Introduction

Follicle‐stimulating hormone (FSH), a key gonadotropin secreted by the anterior pituitary, has traditionally been recognised for its central role in regulating reproductive function through the hypothalamic pituitary gonadal axis [1]. In men, FSH primarily stimulates Sertoli cells in the testes to support spermatogenesis and testicular development [2]. Its biological effects are mediated through binding to the FSH receptor (FSHR), which activates downstream intracellular signalling pathways [3].

Beyond its classical role in reproduction, accumulating evidence suggests that FSH and its receptor may have extragonadal functions, influencing diverse physiological processes such as lipid metabolism, adipogenesis, and systemic energy homeostasis [4]. Immunoreactivity for FSH and its receptor has been identified in several non‐reproductive tissues, including adipose tissue and the pancreas, raising the possibility that FSH may also influence glucose metabolism [5] and may enhance hepatic gluconeogenesis [6]. Emerging evidence suggested that lower FSH levels have been associated with dyslipidemia and type 2 diabetes mellitus (T2DM) in postmenopausal women [7, 8]. Furthermore, FSH has been shown to promote lipid accumulation through FSHR activation in adipose tissue, potentially contributing to adiposity and insulin resistance [9, 10, 11]. It is hypothesised that altered FSH levels may also reflect dysregulation of the HPG axis, which is associated with visceral adiposity, chronic low‐grade inflammation, and impaired insulin signalling, key mechanisms in the pathogenesis of T2DM [12, 13, 14, 15, 16].

In ageing men, FSH levels tend to rise, while testosterone concentrations decline (8). However, while most studies on FSH and metabolic health have focused on women, limited data exist on the role of FSH in men. A recent systematic review and meta‐analysis of 20 studies reported that serum FSH levels were significantly lower in Asian men with T2DM compared to non‐diabetic controls [17], and still, longitudinal data examining whether lower FSH levels predict the future development of T2DM in men remain scarce.

Given the physiological changes in gonadotropin levels following ageing in men and their potential links to glucose metabolism, further longitudinal studies are needed to clarify the role of FSH in the development of T2DM in men. To address this knowledge gap, we conducted a long‐term, population‐based study to examine the association between serum FSH levels and both the prevalence and incidence of T2DM in men aged 40 and older.

## 2 Methodology

This study was reported in accordance with the Strengthening the Reporting of Observational Studies in Epidemiology (STROBE) guidelines for cohort studies. The completed STROBE checklist is provided as STROBE Statement.

### 2.1 Study Design

In this cohort study, subjects were recruited from among participants of the Tehran Lipid and Glucose Study (TLGS), an ongoing population‐based cohort, initiated in 1998 to investigate the prevalence and risk factors of non‐communicable diseases. In summary, in TLGS, a total of 15,005 individuals, aged ≥ 3 years, were followed at intervals of 3 years, to obtain data on demographics, anthropometric, reproductive, hormonal, and metabolic characteristics, general physical examinations and laboratory measurements. For the purpose of the present study, we used TLGS data from six follow‐ups (1st: 2002–2005, 2nd: 2005–2008, 3rd: 2008–2011, 4th: 2011–2014, 5th: 2014–2018, and 6th: 2018–2021).

At each visit, subjects were assessed for clinical, anthropometric, and biochemical parameters by a trained interviewer. Body weight was measured with participants wearing light clothing, using a digital scale (Seca 707, Seca GmbH) and rounded to the nearest 100 g. Likewise, height was measured without shoes in a standing position and with normal posture of the shoulders with a tape measure. Body mass index (BMI) was calculated using the formula [weight in kilograms (kg) divided by height squared (m^2^)]. Waist circumference was measured with an unstretched tape measure at the level of the umbilicus, without any pressure to the body surface. Hip circumference was measured at the level of the anterior superior iliac spine without any pressure to the body surface. We also measured systolic and diastolic blood pressure twice on the right arm in a seated position using a standard mercury sphygmomanometer after 15 min of rest; then the mean of these measurements was recorded.

Serum FSH level was measured at the first visit, while all other biochemical measurements were conducted at baseline and each follow‐up visit. All blood samples were drawn between 7:00 and 9:00 am after 12 h of overnight fasting; blood analyses were performed at the TLGS research laboratory on the day of blood collection. All sera were stored at −80°C until the time of testing. All FSH measurements were taken simultaneously in the same laboratory and were measured using an immunoradiometric assay (IRMA; Izotop, Budapest, Hungary). Plasma glucose was measured using an enzymatic colourimetric method with glucose oxidase. Serum concentrations of triglyceride (TG) were assayed using glycerol phosphate. Total cholesterol (TC) was assayed using the enzymatic colourimetric method with cholesterol esterase and cholesterol oxidase. Levels of high‐density.

Lipoprotein cholesterol (HDL‐C) was measured after precipitation of the apolipoprotein B (apo B)‐containing lipoproteins with phosphotungstic acid. We used a modified Friedewald to calculate LDL‐C. All metabolic analyses were performed using related kits (Pars Azmon Inc., Tehran, Iran) and a Selecta 2 autoanalyser (Vital Scientific, Spankeren, Netherlands). The intra‐ and inter‐assay coefficients of variation (CVs) were both 2.2% for glucose. For both total and HDL cholesterol, intra‐ and inter‐assay CVs were 0.5% and 2%, respectively. Intra and inter‐assay CVs were 0.6% and 1.6% for TG, respectively.

**FIGURE 1:**
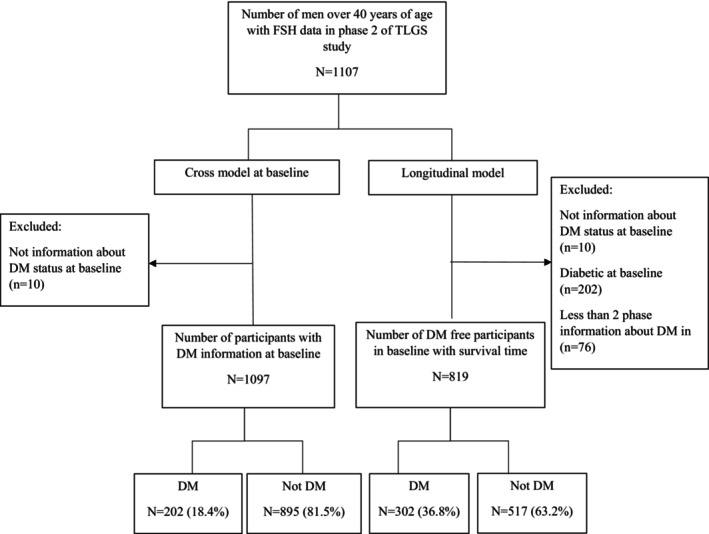
Flow chart of the study.

For the present study, we included all male participants aged ≥ 40 years who met the eligibility criteria and had available FSH measurements at the first follow‐up visit. Two analytical approaches were employed to investigate the association between serum FSH levels and T2DM. First, to examine the association between FSH levels and the prevalence of T2DM, we included all eligible participants regardless of their diabetic status at the first visit, excluding only those with missing data on diabetes status at baseline (*n* = 10). Second, to assess the association between FSH levels and the incidence of T2DM, we excluded participants with missing data on diabetes status at baseline (*n* = 10), those with less than two follow‐up records regarding T2DM (*n* = 76), and those diagnosed with T2DM at baseline (*n* = 202). A detailed overview of the study population selection process is presented in Figure 1.

### 2.2 Terms Definitions

According to American Diabetes Association criteria, T2 DM diabetes was defined as FBS ≥ 7 mmol/L, or 2‐h plasma glucose (OGTT) ≥ 11.1 mmol/L, or using anti‐diabetic drugs [18].

### 2.3 Statistical Analysis

The continuous baseline characteristics of the participants are summarised using either the mean with standard deviation or the median with interquartile range (IQR), depending on whether the distribution is normal or skewed. These characteristics are compared across DM status at baseline and at follow‐ups using the T‐student or the Mann–Whitney U test, as appropriate, based on the normality or skewness of the distribution. The Kolmogorov–Smirnov test is employed to assess the assumption of normality.

To explore the association between baseline FSH levels and prevalent DM at baseline, odds ratios (ORs) along with 95% confidence intervals (CIs) are estimated through logistic regression models. Additionally, Cox proportional‐hazard regression is applied to investigate the predictive effect of baseline FSH levels at baseline of study on the incidence of DM from baseline until the end of follow‐up, using hazard ratios (HRs) and 95% CIs. Participants contribute person‐time from the baseline until a DM diagnosis or, for those without DM, until the date of their last follow‐up clinic visit by the end of the study. Subgroup analyses were performed according to age (< 50, > = 50) and FSH/LH ratio. A high FSH/LH ratio was defined as values above the third quartile (Q3) of the study population distribution. All models are analysed in crude models (Model 1) and adjusted for age and BMI at baseline (Model 2). Further adjustments are made in Model 3 for smoking status, physical activity, family history of DM, HDL, TG, cholesterol, and pre‐DM at baseline. Statistical analyses are performed using SPSS16 and STATA (version 12; STATA Inc., College Station, TX, USA). *p*‐values less than 0.05 are considered statistically significant.

## 3 Results

### 3.1 Baseline Characteristics of Participants

A total of 1097 male participants aged ≥ 40 years were included in the analysis. Table 1 presents the baseline characteristics of the study population overall and stratified by diabetes status at baseline. The mean (SD) age and BMI of participants were 56.0 (6.63) years and 26.68 (3.79) kg/m^2^, respectively. Overall, 45.2% of participants were current smokers, 22.8% had prediabetes, and 26.0% reported a family history of diabetes. Approximately half of the participants (49%) reported a moderate to high level of physical activity. Compared to non‐diabetic participants, those with T2DM were significantly older (60.0 vs. 55.0 years) and had higher BMI (27.74 vs. 26.47 kg/m^2^), systolic blood pressure (130 vs. 121 mmHg), diastolic blood pressure (79.66 vs. 77.17 mmHg), and total cholesterol (205.76 vs. 194.67 mg/dL), respectively (*p* < 0.001 for all).

**TABLE 1:** Baseline characteristics of participants.

Characteristics		Based on the diabetes status at baseline				Based on the diabetes status at follow‐ups			
		Total *N* = 1097	Non‐T2DM *n* = 895	T2DM *n* = 202	*p* *	Total *N* = 819	Non‐T2DM *n* = 517	T2DM *n* = 302	*p* **
Age (Year), Mean (SD)		56 (5.63)	55 (4.63)	60 (5.64)	< 0.001	56 (4.33)	55 (4.68)	56 (4.42)	0.022
BMI (Kg/m^2^), Mean (SD)		26.68 (3.79)	26.47 (3.71)	27.74 (3.96)	< 0.001	26.47 (3.69)	25.98 (3.66)	27.29 (3.59)	0.012
Waist‐to‐hip, Mean (SD)		0.97 (0.93–1.01)	0.96 (0.93–1.00)	0.96 (1.00–1.03)	< 0.001	0.96 (0.93– 1.00)	0.95 (0.92–1.00)	0.97 (0.94–1.01)	< 0.001
SBP (mmHg), Median (IQR)		122 (111–134)	121 (111–131)	130 (118–146)	< 0.001	121 (111–131)	119 (109–131)	123 (115–133)	< 0.001
DBP (mmHg), Mean (SD)		77.63 (11.50)	77.17 (11.34)	79.66 (11.98)	0.006	77 (70.84)	76 (68.83)	79 (71.86)	0.001
TG (mg/dL), Median (IQR)		151 (110–216)	143 (105–204)	188.50 (132.75–260.50)	< 0.001	145 (106–207)	141 (105–202)	155 (113.50–213)	0.066
HDL (mg/dL), Mean (SD)		35 (28.39)	35 (28.39)	34 (28.39)	0.292	35 (28.39)	35 (28.40)	35 (28.39)	0.400
TC (mg/dL), mean (SD)		196.71 (38.88)	194.67 (38.33)	205.76 (40.63)	< 0.001	194.97 (37.43)	192.96 (37.43)	198.42 (37.24)	0.008
Ever Smoker, *N* (%)									0.425
	No	607 (54.8)	484 (54.1)	122 (60.4)	0.103	438 (53.5)	271 (52.4)	167 (55.3)	
	Yes	500 (45.2)	411 (45.9)	80 (39.6)		381 (45.6)	246 (47.6)	135 (44.7)	
Physical activity, *N* (%)					0.769				0.271
	Low	560 (51)	455 (50.8)	105 (52)		416 (50.8)	255 (493)	161 (53.3)	
	High	537 (49)	440 (49.2)	97 (48)		403 (49.2)	262 (50.7)	141 (46.7)	
Family history of DM, *N* (%)					< 0.001				0.031
	No	812 (74)	718 (80.2)	94 (46.5)		651 (79.5)	423 (81.8)	228 (75.5)	
	Yes	285 (26)	177 (19.8)	108 (53.5)		168 (20.5)	94 (18.2)	74 (24.5)	

Abbreviations: T2DM, Type 2 Diabetes Mellitus; BMI, body mass index; SBP, systolic blood pressure; DBP, diastolic blood pressure; HDL, high‐density lipoprotein; TC, Total Cholesterol; FSH, Follicle‐stimulating hormone; DM, Diabetes Mellitus.

* Comparison between the non‐T2DM and T2DM groups who were diagnosed at baseline.

** Comparison between non‐T2DM and T2DM groups who were diagnosed at follow‐ups.

From a total of 819 participants who were free of DM at baseline of the study, 302 (36.8%) men experienced DM at follow‐ups. Table 1 shows the characteristics of these participants at baseline in total and based on DM status at follow‐ups. Compared to non‐diabetic participants, those who developed diabetes at follow‐ups were significantly older (mean age: 55.0 vs. 55.0 years, *p* = 0.022) and had higher BMI (27.29 vs. 25.98 kg/m^2^, *p* = 0.012) and WHR (0.97 vs. 0.95, cm *p* < 0.001).

### 3.2 Associations Between FSH Levels and Prevalence of T2DM at Baseline

Table 2 presents the associations between FSH levels and both the prevalence and incidence of T2DM among participants. At baseline, the prevalence of T2DM was 18.41% (202 out of 1097 participants). The analysis showed no significant association between FSH levels and T2DM prevalence in the crude model (OR: 1.01; 95% CI: 0.99–1.03). The results remained unchanged after adjusting for age and BMI (OR: 1.00; 95% CI: 0.97–1.03), as well as after further adjustment for additional confounders, including smoking status, total cholesterol, physical activity, HDL‐C, triglycerides, prediabetes, and family history of diabetes (OR: 1.00; 95% CI: 0.98–1.03).

**TABLE 2:** Associations between follicle‐stimulating hormone levels and Type 2 Diabetes Mellitus.

		Associations Between FSH levels and prevalence of T2DM at baseline								Associations Between FSH levels and Incidence of T2DM at follow‐ups							
		No. of participants	No. of cases	Crude model OR (95% CI)	*p*	Age and BMI adjusted OR (95% CI)	*p*	Multiple adjusted OR (95% CI)*	*p*	No. of cases	Person‐year	Crude model HR (95% CI)	*p*	Age and BMI adjusted HR (95% CI)	*p*	Multiple adjusted HR (95% CI)*	*p*
Total		1097	202	1.01 (0.99–1.03)	0.241	1.00 (0.97–1.03)	0.725	1.00 (0.98–1.03)	0.493	302	3774.78	0.98 (0.95–1.00)	0.124	**0.97 (0.94–1.00)**	**0.051**	**0.96 (0.93–0.99)**	**0.043**
Subgroup analysis																	
< 50 years		253	21	0.97 (0.85–1.10)	0.667	0.97 (0.85–1.11)	0.728	0.98 (0.85–1.13)	0.828	88	1269.40	0.97 (0.91–1.02)	0.383	0.98 (0.92–1.04)	0.566	0.97 (0.91–1.03)	0.390
> = 50 years		844	181	1.01 (0.98–1.03)	0.465	1.00 (0.98–1.03)	0.646	1.01 (0.98–1.04)	0.430	214	2505.38	0.97 (0.94–1.00)	0.121	0.97 (0.94–1.00)	0.081	**0.96 (0.93–0.99)**	**0.047**
High FSH/LH**		275	60	0.99 (0.95–1.02)	0.577	0.98 (0.95–1.02)	0.492	0.99 (0.95–1.02)	0.639	70	874.97	0.96 (0.92–1.00)	0.076	**0.95 (0.92–0.99)**	**0.042**	**0.94 (0.90–0.99)**	**0.024**

*Note:* Bold values indicate statistically significant.

* Adjusted by age, BMI, smoking status, cholesterol, physical activity, HDL, TG, pre‐DM, and family history of DM.

** Participants were categorised as having a high FSH/LH ratio if their ratio was in the highest quartile (Q3 or above).

Subgroup analyses by age (< 50 and ≥ 50 years) also showed no significant associations between FSH levels and T2DM prevalence across any of the models.

### 3.3 Associations Between FSH Levels and Incidence of T2DM During Follow‐Up

Over a total of 3774.78 person‐years of follow‐up, 302 new cases of T2DM were identified. In the crude model, FSH levels were not significantly associated with the risk of developing T2DM (HR: 0.98; 95% CI: 0.95–1.00). After adjustment for age and BMI, the association approached statistical significance (HR: 0.97; 95% CI: 0.94–1.00). In the fully adjusted model, which controlled for multiple potential confounders, a statistically significant inverse association was observed (HR: 0.96; 95% CI: 0.93–0.99), indicating that higher FSH levels were associated with a lower risk of developing T2DM.

### 3.4 Subgroup Analysis Based on Age and High FSH/LH Ratio

Subgroup analyses by age group revealed no significant associations among participants aged < 50 years (88 cases, 1269.40 person‐years) across all models. Among participants aged ≥ 50 years (214 cases, 2505.38 person‐years), a statistically significant inverse association was found in the fully adjusted model (HR: 0.96; 95% CI: 0.93–0.99), indicating that higher FSH levels were independently associated with a lower risk of developing T2DM in older men.

Subgroup analyses by high FSH/LH ratio showed no significant associations between FSH and the prevalence of T2DM at baseline across crude and adjusted models. While statistically significant inverse associations were found in the age and BMI‐adjusted model (HR: 0.95; 95% CI: 0.92–0.99) and fully adjusted model (HR: 0.94; 0.95% CI: 0.90–0.99), indicating that higher FSH levels were associated with a lower risk of developing T2DM in men with high FSH/LH.

## 4 Discussion

In this long‐term, prospective population‐based study, we observed that while serum FSH levels were not associated with the prevalence of T2DM at baseline, higher FSH levels were significantly and independently associated with a lower risk of developing T2DM over time. This inverse association was particularly evident among men aged ≥ 50 years, even after adjusting for key demographic and metabolic confounders. These findings suggest that elevated FSH levels may play a protective role in the development of T2DM in older men. This novel finding, to our knowledge, the first prospective evidence in a male cohort aged over 40 years, suggests a potential protective role of elevated FSH levels against T2DM development in older men, warranting further exploration into its clinical implications.

T2DM remains a leading risk factor for cardiovascular disease, with its global prevalence continuing to rise, posing a significant public health burden [19, 20, 21, 22]. It is well documented that a wide range of factors, including age, sex, genetic predisposition, ethnicity, obesity, and family history, are known to influence the development of T2DM [23, 24, 25, 26]; nevertheless, there are still unknown factors that contribute to DM pathogenesis. However, emerging evidence suggests that hormonal factors, particularly gonadotropins like FSH, may also play a role in metabolic health [27, 28, 29, 30].

FSH, a glycoprotein peptide hormone synthesised by the anterior pituitary, can affect both the reproductive and non‐reproductive systems [31, 32]. Growing evidence indicates that FSH may also influence glucose metabolism, adiposity, and inflammatory status. Our finding of an inverse association between FSH levels and T2DM incidence aligns with prior studies observing inverse associations of FSH levels with T2DM in postmenopausal women (28) or lower levels of FSH in men with T2DM (15).

While the underlying mechanisms of association between FSH and glucose intolerance are not yet fully clarified, it is hypothesised that alterations in insulin sensitivity and changes in lipid metabolism, body composition, and inflammatory pathways may mediate these associations [31, 33, 34]. Recent evidence showed that FSH receptors have been identified in extragonadal tissues, including pancreatic cells and adipose tissue [5, 35]. Chu et al. identified FSH in the rat pancreas, reporting co‐expression of FSH and FSH receptors in islet cells, suggesting a potential direct effect on glycemic regulation [5]. Additionally, it is shown that FSH may modulate lipid metabolism and promote adipocyte differentiation through activation of FSH receptors expressed on adipose tissue, which may contribute to increased adiposity and systemic insulin resistance [36, 37]. As such, lower FSH levels may reflect a disrupted hypothalamic–pituitary–gonadal (HPG) axis, which has been associated with altered energy balance, increased visceral fat accumulation, and pro‐inflammatory states, all of which are established contributors to insulin resistance and glucose dysregulation [38].

As such, another important mechanistic consideration is the potential confounding role of obesity‐related leptin resistance on hypothalamic–pituitary‐gonadal (HPG) axis function. Hyperleptinemia, a hallmark of visceral adiposity and metabolic dysfunction, has been shown to suppress gonadotropin‐releasing hormone (GnRH) pulsatility at the hypothalamic level, thereby attenuating pituitary FSH secretion and producing a state of central hypogonadism that mirrors the low‐FSH phenotype observed in metabolically compromised individuals [39, 40]. In a recently published study, Li et al. demonstrated in a cross‐sectional study of young and middle‐aged men that obesity‐associated secondary hypogonadism was characterised by significantly reduced FSH levels, attributable to central HPG axis inhibition driven by hyperleptinemia, underscoring that low FSH may function as an integrated biomarker of metabolically unhealthy obesity rather than an independent hormonal determinant of metabolic risk [41]. Although our multivariable models were adjusted for BMI, a conventional surrogate of adiposity, BMI does not fully capture visceral fat distribution or the degree of leptin resistance, which may have introduced residual confounding.

Interestingly, our findings suggest that the inverse association between FSH levels and incident T2DM is more pronounced in men aged 50 years and older. One possible explanation is that hormonal changes accompanying ageing, including declining testosterone and alterations in oestrogen metabolism, may interact with FSH in ways that influence glucose regulation and insulin sensitivity [42]. Additionally, ageing is associated with changes in body composition, including increased visceral adiposity and inflammation, which may amplify the metabolic effects of altered gonadotropin levels [43, 44]. These age‐related physiological shifts could enhance the biological relevance of FSH in glucose homeostasis and explain why its association with T2DM risk is more evident in this age group [45].

Additionally, we found that, same as higher FSH levels alone, in advanced age, high FSH/LH ratios have a protective effect on incident T2DM that was confined to men with high FSH/LH ratios. This pattern suggests that the relative gonadotropin milieu, not only absolute FSH concentrations, may be metabolically relevant. An elevated FSH/LH ratio is characteristic of the ageing male hormonal axis, reflecting differential changes in pituitary gonadotropin secretion as Leydig cell function declines with age [46, 47]. In this context, a higher FSH/LH ratio may serve as an integrated marker of a specific endocrine state in which FSH‐mediated signalling predominates, potentially exerting protective effects on glucose metabolism through pathways that are attenuated when LH‐driven androgen production remains comparatively robust [48, 49]. These findings are consistent with emerging evidence indicating that FSH exerts direct metabolic actions beyond its classical reproductive role, including modulation of adipose tissue metabolism and insulin sensitivity [49, 50].

An important issue in our findings is whether the inverse association between FSH and incident T2DM reflects an independent hormonal effect or primarily mirrors underlying metabolic dysregulation. In our cohort, the association between higher FSH and lower diabetes risk remained statistically significant after comprehensive adjustment for major metabolic risk factors, which may indicate that FSH is not simply a surrogate marker of conventional metabolic risk. Nevertheless, metabolic and hormonal systems are tightly interrelated and likely act in concert rather than independently. Metabolic disturbances such as visceral adiposity, insulin resistance, and dyslipidemia can suppress hypothalamic GnRH pulsatility through mechanisms involving hyperleptinemia and inflammatory signalling, leading to reduced pituitary gonadotropin secretion and a functional hypogonadal state [51, 52]. Conversely, alterations in gonadotropin signalling may also influence metabolic regulation via extragonadal pathways, including adipose tissue and pancreatic function [53]. Within this bidirectional framework, low FSH may partly reflect accumulated metabolic burden, while higher FSH, particularly in older men and those with elevated FSH/LH ratios, may indicate a more preserved hypothalamic–pituitary–gonadal axis and a relatively favourable metabolic state. Importantly, the persistence of the association after metabolic adjustment, together with our FSH/LH ratio findings, suggests that the gonadotropin milieu provides additional prognostic information beyond standard metabolic markers.

Strengths of our study include its prospective design, large sample size, and extensive adjustment for potential confounders, alongside age‐stratified analyses that revealed the age‐specific effect. However, some limitations should be acknowledged. FSH was measured at a single time point, which limits our ability to evaluate longitudinal changes or intra‐individual variability over time. As such, although we adjusted for a wide range of covariates, the possibility of residual confounding cannot be entirely excluded. Another limitation is that testosterone measurements were available for only a small subset of participants (~17%), precluding their inclusion in the main analyses. Similarly, leptin levels were not measured in our dataset, preventing us from evaluating potential effect modification by leptin or adjusting for leptin resistance. Further, our study included only men, and results may not be generalisable to women or to ethnically diverse populations. Lastly, we did not assess levels of other sex hormones (such as luteinising hormone, estradiol, or testosterone), which may have provided additional insight into the hormonal milieu influencing T2DM risk.

## 5 Conclusion

In conclusion, higher serum FSH levels were independently associated with a reduced risk of incident T2DM in men, particularly among those aged ≥ 50 years. These findings suggest a potential protective metabolic role of FSH in older men. Further research into the non‐reproductive roles of FSH is warranted.

## Author Contributions

F.R.T., F.A., and S.B.‐G. Conception or design. F.R.T., M.M., F.F., F.A., and S.B.‐G. Acquisition, analysis, or interpretation of data. M.M., S.B.‐G., and F.R.T. Drafting the work or revising. All authors: final approval of the manuscript.

## Funding

This work was supported by the Research Institute for Endocrine Sciences, Shahid Beheshti University of Medical Sciences, 1‐43008667, Nord University OA fund for publication.

## Ethics Statement

The current study was approved by the ethical review board of the Research Institute for Endocrine Sciences (IR. SBMU.ENDOCRINE.REC1402.104).

## Consent

Informed consent was obtained from all participants involved in the study.

## Conflicts of Interest

The authors declare no conflicts of interest.

## Supporting information


**Data S1:**: STROBE Statement—Checklist of items that should be included in reports of *cohort studies*.

## Data Availability

The data that support the findings of this study are available on request from the corresponding author. The data are not publicly available due to privacy or ethical restrictions.
