# Supplementary material for: Association Between Serum Follicle‐Stimulating Hormone Levels and Risk of Diabetes in Middle‐Aged Men: A Long‐Term Population‐Based Longitudinal Study
Source: Endocrinol Diabetes Metab. 2026 Jul 6;9(4):e70262. doi: 10.1002/edm2.70262 (PMC13334371; doi:10.1002/edm2.70262)
Supplement: Supplementary file 1 — Data S1:: STROBE Statement—Checklist of items that should be included in reports of cohort studies. [file EDM2-9-e70262-s001.docx]

STROBE Statement—Checklist of items that should be included in reports of ***cohort studies***

|  | | Item No | Recommendation | Reported on section |
| --- | --- | --- | --- | --- |
| **Title and abstract** | | 1 | (*a*) Indicate the study’s design with a commonly used term in the title or the abstract | Title + Abstract |
|  |  |  | (*b*) Provide in the abstract an informative and balanced summary of what was done and what was found | Abstract |
| Introduction | | | | |
| Background/rationale | | 2 | Explain the scientific background and rationale for the investigation being reported | Section 1 (Introduction) |
| Objectives | | 3 | State specific objectives, including any prespecified hypotheses | Section 1 (Introduction)  (last paragraph) |
| Methods | | | | |
| Study design | | 4 | Present key elements of study design early in the paper | Section 2.1 |
| Setting | | 5 | Describe the setting, locations, and relevant dates, including periods of recruitment, exposure, follow-up, and data collection | Section 2.1 |
| Participants | | 6 | (*a*) Give the eligibility criteria, and the sources and methods of selection of participants. Describe methods of follow-up | Section 2.1 (incl. Figure 1) |
|  |  |  | (*b*) For matched studies, give matching criteria and number of exposed and unexposed | N/A (Not a matched study) |
| Variables | | 7 | Clearly define all outcomes, exposures, predictors, potential confounders, and effect modifiers. Give diagnostic criteria, if applicable | Section 2.2 + 2.3 |
| Data sources/ measurement | | 8* | For each variable of interest, give sources of data and details of methods of assessment (measurement). Describe comparability of assessment methods if there is more than one group | Section 2.1 |
| Bias | | 9 | Describe any efforts to address potential sources of bias | Section 2.3 |
| Study size | | 10 | Explain how the study size was arrived at | Section 2.3 |
| Quantitative variables | | 11 | Explain how quantitative variables were handled in the analyses. If applicable, describe which groupings were chosen and why | Section 2.3 + Table 2 (subgroups) |
| Statistical methods | | 12 | (*a*) Describe all statistical methods, including those used to control for confounding | Section 2.3 |
|  |  |  | (*b*) Describe any methods used to examine subgroups and interactions | Section 2.3 + 3.4 |
|  |  |  | (*c*) Explain how missing data were addressed | Section 2.3 |
|  |  |  | (*d*) If applicable, explain how loss to follow-up was addressed | Section 2.3 |
|  |  |  | (*e*) Describe any sensitivity analyses | Section 2.3 |
| Results | | | |  |
| Participants | | 13* | (a) Report numbers of individuals at each stage of study—eg numbers potentially eligible, examined for eligibility, confirmed eligible, included in the study, completing follow-up, and analysed | Section 2.1 + Figure 1 |
|  |  |  | (b) Give reasons for non-participation at each stage | N/A |
|  |  |  | (c) Consider use of a flow diagram | Figure 1 |
| Descriptive data | | 14* | (a) Give characteristics of study participants (eg demographic, clinical, social) and information on exposures and potential confounders | Table 1 |
|  |  |  | (b) Indicate number of participants with missing data for each variable of interest | N/A |
|  |  |  | (c) Summarise follow-up time (eg, average and total amount) | Section 3.3 |
| Outcome data | | 15* | Report numbers of outcome events or summary measures over time | Section 3.1 + 3.3 |
| Main results | 16 | (*a*) Give unadjusted estimates and, if applicable, confounder-adjusted estimates and their precision (eg, 95% confidence interval). Make clear which confounders were adjusted for and why they were included | | Table 2 |
|  |  | (*b*) Report category boundaries when continuous variables were categorized | | Yes (age <50/≥50, high FSH/LH) |
|  |  | (*c*) If relevant, consider translating estimates of relative risk into absolute risk for a meaningful time period | | Not done |
| Other analyses | 17 | Report other analyses done—eg analyses of subgroups and interactions, and sensitivity analyses | | Section 3.4 |
| Discussion | | | | |
| Key results | 18 | Summarise key results with reference to study objectives | | Section 4 (first paragraph) |
| Limitations | 19 | Discuss limitations of the study, taking into account sources of potential bias or imprecision. Discuss both direction and magnitude of any potential bias | | Section 4 (limitations paragraph) |
| Interpretation | 20 | Give a cautious overall interpretation of results considering objectives, limitations, multiplicity of analyses, results from similar studies, and other relevant evidence | | Section 4 |
| Generalisability | 21 | Discuss the generalisability (external validity) of the study results | | Section 4 (limitations) |
| Other information | | | | |
| Funding | 22 | Give the source of funding and the role of the funders for the present study and, if applicable, for the original study on which the present article is based | | Funding section |
